# Dysentery Due to Y Bacillus

**Published:** 1915-06

**Authors:** 


**Dysentery Due to Y Bacillus.** R. H. Norgate and I. Walker Hall of Bristol, Bristol Medico-Chi. Jour., Mch. 1915. The initial case was a man who had been working with colonial troops on Salisbury Plain. 40 cases developed, five fatal. Nurses, visitors, persons in homes visited by nurses and discharged patients were included.

